# Age‐specific sensitivity analysis of stable, stochastic and transient growth for stage‐classified populations

**DOI:** 10.1002/ece3.9561

**Published:** 2022-12-19

**Authors:** Stefano Giaimo, Arne Traulsen

**Affiliations:** ^1^ Department of Evolutionary Theory Max Planck Institute for Evolutionary Biology Plön Germany

**Keywords:** aging, elasticity analysis, population growth, senescence, sensitivity analysis, stage, stochastic growth rate, transient growth

## Abstract

Sensitivity analysis in ecology and evolution is a valuable guide to rank demographic parameters depending on their relevance to population growth. Here, we propose a method to make the sensitivity analysis of population growth for matrix models solely classified by stage more fine‐grained by considering the effect of age‐specific parameters. The method applies to stable population growth, the stochastic growth rate, and transient growth. The method yields expressions for the sensitivity of stable population growth to age‐specific survival and fecundity from which general properties are derived about the pattern of age‐specific selective forces molding senescence in stage‐classified populations.

## Graphical abstract

Obtain the sensitivity of population growth (stable, transient, or stochastic) to age‐specific perturbations in stage‐classified populations by simple modifications of formulas for the age‐independent case. These modified formulas also illuminate the pattern of selective forces molding senescence in stage‐classified populations.

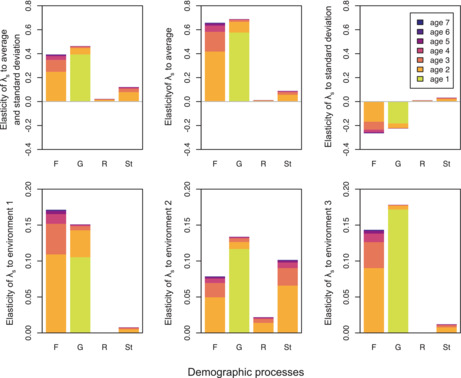

## 1 INTRODUCTION

Matrix models are a widely used tool for demographic analysis. They require individuals in the population to be grouped into separate classes and the individual dynamics through the classes to be quantified (Caswell, 2001). A basic application of matrix models is demographic projection, which consists in updating population abundances starting from a given initial state. A salient feature of these models is their being amenable to the sensitivity analysis of virtually any function, for example, the population growth rate, to the demographic parameters they contain (Caswell, 2019).

Age is a classic descriptor for individual classification. It is at the basis of the Leslie matrix model (Keyfitz & Caswell, 2005), where individuals are classified into separate age classes and their age‐specific survival and fecundity are quantified. Stage is another classic descriptor. A stage may be a size class (Calvo & Horvitz, 1990; Hoffmann, 1999; Pfeifer et al., 2006), a developmental phase (Kaneko & Takada, 2014), an occupied territory (Stephens et al., 2002), the position of the individual within a network (Giaimo et al., 2018), or any other individual state variable. More sophisticated demographic classifications are possible. For example, lately there has been a rise in the need of classifying individuals by both age and stage (Caswell, 2012; Caswell et al., 2018; Steiner et al., 2014) or through multiple descriptors of individual state (Roth & Caswell, 2016).

However, in matrix models purely based on stage, which are the focus of the present work, age is not absent. It merely is implicit (Cochran & Ellner, 1992). With each update of stage abundances, individuals either die or survive while getting one time unit older. Leveraging this ineluctable demographic fact, methods have been proposed to extract from stage‐classified matrix models a variety of age‐related quantities. These include, for example, stage‐specific life expectancy, average age at parenthood, or average age‐specific fecundity (Caswell, 2001, ch. 5, 2009; Cochran & Ellner, 1992; Horvitz & Tuljapurkar, 2008; Tuljapurkar & Horvitz, 2006). The reason that age‐from‐stage methods exist is that they are a natural response to the need of analyzing along the “age” dimension the numerous matrix models that have been already constructed exclusively on the basis of stage and not of age. Moreover, matrix models only based on stage are arguably more prevalent than matrix models based on both age and stage, as age × stage models require more data for their parameterization and these additional data may not always be available or easy to collect. The presence of environmental variability may further exacerbate this unbalance as multiple matrix models need to be parameterized for the same population when the environment fluctuates.

A form of age‐from‐stage method is the sensitivity analysis of population growth for stage‐classified populations with respect to age‐specific perturbations. In a more general work on age × stage matrix models, Caswell (2012) proposed a way to perform this analysis for the stable growth rate of matrix models of populations classified by stage and living under constant environmental conditions. Caswell's method is based on the construction, from the stage‐based matrix model, of another, larger matrix model that includes both age and stage classification to then perform age‐specific sensitivity analysis of population growth using matrix calculus on the derived model. An application of this method is the quantification of selection gradients on age‐specific mortality and fecundity from matrix models of populations classified solely by stage (Caswell, 2012; Caswell & Salguero‐Gómez, 2013).

The general framework for the analysis of age × stage matrix models upon which Caswell's method for the analysis of stage‐based models relies is very general and so is the matrix calculus upon which it is based (Caswell, 2019; Caswell et al., 2018). This framework covers way more than the sensitivity analysis of population growth. It is also possible to envisage that such framework could be extended to capture the sensitivity analysis of transient population growth and long‐run population growth to age‐specific parameters for stage‐classified models under environmental stochasticity. Notably, an invitation to extend the framework for the analysis of age × stage matrix models to the case of changing environments is contained in Caswell et al. (2018, p. 581). This appears as a potentially important extension, as environmental fluctuations are a very common condition for most populations (Bernhardt et al., 2020). But, to the best of our knowledge, there has been so far no visible progress along the front of explicitly applying age‐specific sensitivity analysis of the growth rate for stage‐classified populations under environmental stochasticity.

In this work, we do note embark into the broad endeavor of extending the general analysis of age × stage matrix models. We do, however, go in the direction of expanding along the “age” dimension the scope of sensitivity analysis of population growth for matrix models solely classified by stage. In doing so, we hope to contribute to the mentioned need of analyzing the already available matrix models that happen to be based on stage only, and not on age. Here, we propose a method that is alternative to, and larger in scope than, that proposed by Caswell (2012) for the sensitivity analysis of population growth in models only classified by stage with respect to age‐specific perturbations. Our method is alternative to that of Caswell (2012) because we do not require the separate construction and analysis of an age × stage matrix model (although a formal equivalence between the two methods is shown in the Appendix A.2). Our method is larger in scope because it directly applies to stable population growth in a constant environment as well as to transient and long‐run population growth under environmental stochasticity. We propose two applications of our method to evidence what we believe are some of its advantages. The first application is the analysis of age‐specific selective forces molding senescence in stage‐classified populations. We show how some properties of these forces, which were made observable in Caswell and Salguero‐Gómez (2013), can now be predicted analytically via our method. The second application is the decomposition of the elasticities of transient and stochastic growth into age‐specific components. This application shows how our method can yield a fine‐grained version of the sensitivity analysis of population growth for stage‐classified models.

Finally, we would like to stress that our method for age‐specific sensitivity analysis only applies to matrix models structured solely by stage. The method has no say on matrix models that are structured by both age and stage.

## 2 SENSITIVITY ANALYSIS OF POPULATION GROWTH

Before starting with the analysis, we stipulate a convention. In the main text, we prefer to limit ourselves to account for the influence of changes in a single parameter on population growth. For this reason, we find convenient to adopt the formalism according to which the derivative ∂A/∂θ of a matrix $A=\left(a_{i,j}\right)$ with respect to a parameter θ is a matrix of the same dimensions as A and the $\left(i,j\right)$ entry of ∂A/∂θ is $\partial a_{i,j}/\partial \theta$. Similarly, the derivative ∂x/∂θ of a vector $x={\left(x_{1},x_{2},\ldots ,x_{n}\right)}^{T}$, where the superscript Τ indicates transposition, is the vector $\partial x/\partial \theta ={\left(\partial x_{1}/\partial \theta ,\partial x_{2}/\partial \theta ,\ldots ,\partial x_{n}/\partial \theta \right)}^{Τ}$. This is the formalism in Caswell (2001). However, it is sometimes useful to consider the effect on population growth of multiple parameters. While this can be done by iterating one‐parameter formulas for each of the parameters, another possibility is to do all this at once. In Section A.7 of the Appendix, we generalize results from the main text to account for the effect on growth of changes in multiple age‐specific parameters. Therein, we describe and use a different, more apt formalism for the derivative of a vector or a matrix with respect to a vector of parameters that follows the conventions of the matrix calculus introduced to ecology by Caswell (2007, 2009). We assume throughout that the matrix model denoted with A, or $A\left(t\right)$ in the time‐varying case, is classified solely by stage.

### 2.1 No age specificity

Here, we recall known results from age‐independent sensitivity analysis of population growth. A column vector $n\left(t\right)$ describes stage‐specific abundances in the population at time t. The component i of this vector is the population abundance in stage i at time t. In one time unit, each individual in stage j contributes $a_{i,j}$ individuals to stage i. When the environment is constant, this contribution is constant too. The dynamics of $n\left(t\right)$ are then determined by the projection matrix $A=\left(a_{i,j}\right)$, which is assumed to be classified by stage, as

(1) $$n\left(t+1\right)=\text{An}\left(t\right).$$

At demographic stability, the total population size grows every time step by a factor corresponding to the dominant eigenvalue λ of A and the relative fraction in stage j remains constant and equal to the component $w_{j}$ of the right dominant eigenvector w of A. This eigenvector is assumed to be normalized so that its components add up to 1. The left dominant eigenvector v contains reproductive values, which capture the relative importance of individuals to future population growth depending on their stage.

Suppose A depends on some parameter θ with current value $\theta^{*}$. A classic result by Caswell (1978) is that the sensitivity of stable population growth on the log scale, lnλ, to this parameter is

(2) $$\frac{\partial \ln\lambda }{\partial \theta }=\frac{v^{Τ}\frac{\partial A}{\partial \theta }w}{\lambda v^{Τ}w},$$

where the derivative should be understood as evaluated at $\theta =\theta^{*}$.

An analogous result holds when the environment can be in different states that affect the demography. Assume transitions among environmental states obey an ergodic time‐homogeneous Markov chain and to each environmental state there corresponds a projection matrix. The update of stage‐specific abundances then takes the form

(3) $$n\left(t+1\right)=A\left(t\right)n\left(t\right),$$

where $A\left(t\right)$ is the projection matrix for the environmental state at t. As most authors, we assume that in the long run and independently of the initial stage distribution, population growth is characterized by the stochastic growth rate $\ln\lambda_{s}$ (which is valid under the assumptions discussed by Cohen (1977a, 1977b); Tuljapurkar (1990)),

(4) $$\ln\lambda_{s}=\lim_{t\to \infty }\frac{1}{t}\ln\left[\frac{N\left(t\right)}{N\left(0\right)}\right],$$

where $N\left(t\right)$ is population size at t.

Tuljapurkar (1990) pioneered sensitivity analysis of the stochastic growth rate. He defined three main quantities: the time‐specific stage distribution $w\left(t\right)$, the time‐specific reproductive value vector $v\left(t\right)$, which are vectors scaled so that the components of each add up to 1 (for the computation of these quantities see Section A.3 of the Appendix), and the time‐specific growth

(5) $$\lambda_{t}=\frac{e^{Τ}n\left(t+1\right)}{e^{Τ}n\left(t\right)},$$

where e is a vector of 1s. The quantity $\lambda_{t}$ is the factor by which population size changes between t and t+1. Suppose that the matrices for demographic projection depend on some parameter θ. Expanding upon the original analysis of Tuljapurkar (1990), Caswell (2005) showed that the sensitivity of long‐run population growth to a parameter θ (with current value $\theta^{*}$) upon which some demographic rates depend is

(6) $$\frac{\partial \ln\lambda_{s}}{\partial \theta }=\lim_{L\to \infty }\frac{1}{L}\sum_{t=0}^{L-1}\frac{v^{Τ}\left(t+1\right)\frac{\partial A\left(t\right)}{\partial \theta }w\left(t\right)}{\lambda_{t}v^{Τ}\left(t+1\right)w\left(t+1\right)},$$

which is a stochastic analog of Equation 2 and where the derivative should be understood as evaluated at $\theta =\theta^{*}$. The chief approach to estimate $\partial \ln\lambda_{s}/\partial \theta$ is via extensive stochastic simulations of the demographic process (Caswell, 2001; Morris & Doak, 2002).

Finally, time‐specific growth (sometimes called the transient population growth rate) $\ln\lambda_{t}$ is susceptible of sensitivity analysis too. Combining and differentiating Equations 3 and 5, Caswell (2007, eq. 43) found that

(7) $$\frac{\partial \ln\lambda_{t}}{\partial \theta }=\frac{e^{Τ}}{N\left(t+1\right)}\frac{\partial n\left(t+1\right)}{\partial \theta }-\frac{e^{Τ}}{N\left(t\right)}\frac{\partial n\left(t\right)}{\partial \theta },$$

where the vectors $\partial n\left(t\right)/\partial \theta$ are computed via the recursion

(8) $$\frac{\partial n\left(t+1\right)}{\partial \theta }=\frac{\partial A\left(t\right)}{\partial \theta }n\left(t\right)+A\left(t\right)\frac{\partial n\left(t\right)}{\partial \theta },$$

from given initial vectors $n\left(0\right)$ and $\partial n\left(0\right)/\partial \theta$. Caswell (2007) gives information on how to set the latter vector. A particularly relevant case is when θ has no effect on the initial population and $\partial n\left(0\right)/\partial \theta$ is the zero vector. The derivatives in Equations 7 and 8 should be understood as evaluated at θ's current value.

### 2.2 Adding age specificity

Suppose we are given a stage‐classified matrix model A. In performing sensitivity analysis of the stable population growth for this matrix, Equation 2 presupposes that, when θ changes, all individuals with demographic rates that depend on θ will be equally affected regardless of the age of these individuals. This is because the entries of A express stage‐specific quantities. Although the given model is classified by stage, changes in the population are theoretically conceivable so that they only affects individuals of age j (Caswell, 2012, p. 408). And we may want to find out the resulting effect on population growth. Supposing that no additional age‐specific data are available or can be collected for the target population, to analyze the age‐specific sensitivity of population growth one can only try to squeeze information out of the given stage‐classified model. To get the relevant information for this purpose, Caswell (2012) proposed the construction of an age × stage matrix model from the entries of the stage‐classified model. Age‐specific sensitivity analysis can then be performed on the obtained age × stage model and not anymore on the original stage‐classified model. Here, we propose an alternative way of addressing the problem of getting age‐specific sensitivity of population growth from models that were constructed using a stage‐based classification. We deem our proposal alternative because it does not pass through the construction of an age × stage matrix model. (However, we can prove an essential equivalence with Caswell's approach, see Section A.2 of the Appendix.) Our proposal in essence consists in only slightly modifying the results of the age‐independent sensitivity analysis reviewed in the previous section to make them age‐specific.

We start from the case of a constant environment. To emphasize that we now analyze a scenario characterized by age‐specific effects, we write the parameter as $\theta_{j}$. Since A is assumed classified by stage, it is very hard to envisage how we could, if ever, introduce in it a perturbation parameter for age‐specific effects. The matrix A governs the demography of individuals depending on their stage and independently of their age. Modifying entries of A implies modifying demographics in an age‐independent fashion. To solve this problem, we propose the following construction. First, rewrite Equation 1 as

(9) $$n\left(t+1\right)=\sum_{k=1}^{\infty }A_{k}n_{k}\left(t\right),$$

where $A_{k}=A$ for k=1,2,…, while $n_{k}\left(t\right)$ contains stage‐specific abundances of individuals aged k, that is, the i component of this vector is the number of individuals in stage i and of age k at t and

$$n\left(t\right)=\sum_{k=1}^{\infty }n_{k}\left(t\right).$$

Equation 9 separately projects each age class using the same demographic rates. Using the construction in Equation 9, we can specifically target the demographic rates of individuals aged j by supposing that the matrix $A_{j}$ depends on some parameter $\theta_{j}$ with current value $\theta_{j}^{*}$. We then rewrite Equation 9 as

(10) $$n\left(t+1\right)=\sum_{\begin{array}{c} k=1\\ k\neq j \end{array}}^{\infty }A_{k}n_{k}\left(t\right)+A_{j}\left(\theta_{j}^{*}\right)n_{j}\left(t\right),$$

where all matrices are still identical with one another but we have singled out $A_{j}$ to highlight its dependence on a parameter upon which the other matrices do not depend.

Next, we note that Equation 2 makes the mechanism through which a change in θ affects stable population growth transparent. The quantity $a_{i,j}$ is the demographic contribution of an individual in stage j to stage i per time step. The $\left(i,j\right)$‐entry of the matrix ∂A/∂θ is the sensitivity of $a_{i,j}$ to θ. In Equation 2, this sensitivity is weighted to contribute toward ∂lnλ/∂θ. The weight is the product $v_{i}w_{j}$, which accounts both for the relative number $w_{j}$ of individuals whose demographic rates are directly dependent on θ, that is, the individuals in stage j, and for the relevance, given by the reproductive value $v_{i}$, to population growth of a change that alters the demographic influx to stage i. Equation 10 and the logic behind Caswell's result suggest a straightforward way of getting an expression for the age‐specific sensitivity of lnλ. We should modify Equation 2 in two ways: we should substitute the matrix ∂A/∂θ of sensitivities of age‐independent demographic rates with the matrix $\partial A_{j}/\partial \theta_{j}$ of age‐specific sensitivities and we should substitute the stable stage distribution w with the fraction $w_{j}$ of the stable stage distribution aged j. The i component of $w_{j}$ is the stable population fraction in stage i and of age j. Thus

(11) $$\frac{\partial \ln\lambda }{\partial \theta_{j}}=\frac{v^{Τ}\frac{\partial A_{j}}{\partial \theta_{j}}w_{j}}{\lambda v^{Τ}w},$$

where, as in the corresponding age‐independent formula, the derivative is evaluated at $\theta_{j}$'s current value. In this way, the sensitivity of lnλ specifically accounts for the relative size of that part of the population that has demographic rates that depend on $\theta_{j}$. A formal derivation of Equation 11 is in Section A.1 of the Appendix. An essential equivalence with the approach of Caswell (2012) is shown in Section A.2 of the Appendix.

The reasoning leading to Equation 11 smoothly extends to the case of environmental variability. Equation 3 for projection under a changing environment can be decomposed into the projection of separate age classes as Equation 1. The only difference is that projection matrices and stage distribution now depend on time t. Accordingly, an age‐specific version of the sensitivity formula in Equation 6 is

(12) $$\frac{\partial \ln\lambda_{s}}{\partial \theta_{j}}=\lim_{L\to \infty }\frac{1}{L}\sum_{t=0}^{L-1}\frac{v^{Τ}\left(t+1\right)\frac{\partial A_{j}\left(t\right)}{\partial \theta_{j}}w_{j}\left(t\right)}{\lambda_{t}v^{Τ}\left(t+1\right)w\left(t+1\right)},$$

A more formal derivation of this equation is in Section A.3 of the Appendix.

Finally, the same strategy yields the age‐specific sensitivity of time‐specific (or transient) growth,

(13) $$\frac{\partial \ln\lambda_{t}}{\partial \theta_{j}}=\frac{e^{Τ}}{N\left(t+1\right)}\frac{\partial n\left(t+1\right)}{\partial \theta_{j}}-\frac{e^{Τ}}{N\left(t\right)}\frac{\partial n\left(t\right)}{\partial \theta_{j}},$$

where the vectors $\partial n\left(t\right)/\partial \theta_{j}$ in Equation 13 are computed via the recursion

(14) $$\frac{\partial n\left(t+1\right)}{\partial \theta_{j}}=\frac{\partial A_{j}\left(t\right)}{\partial \theta_{j}}n_{j}\left(t\right)+A\left(t\right)\frac{\partial n\left(t\right)}{\partial \theta_{j}},$$

from given initial vectors $n\left(0\right)$ and $\partial n\left(0\right)/\partial \theta_{j}$. A derivation of Equation 14 is in Section A.4 of the Appendix.

### 2.3 Age‐specific stage distribution

To obtain $w_{j}\left(t\right)$ in Equation 12, we represent the projection matrix as a sum $A\left(t\right)=U\left(t\right)+F\left(t\right)$, where $U\left(t\right)=\left(u_{i,j}\left(t\right)\right)$ is the transition matrix and $F\left(t\right)=\left(f_{i,j}\left(t\right)\right)$ is the fecundity matrix. The quantity $u_{i,j}\left(t\right)$ is the probability that an individual in stage j at t is in stage i at t+1, while $f_{i,j}\left(t\right)$ is the number of new recruits in stage i at t+1 per individual in stage j at t. Age 1 is assigned to new recruits at their first census. Age is updated at demographic projection so that an individual of age j at t is of age j+1 at t+1. Thus, the stage distribution aged j at time t≥j is

(15) $$\begin{array}{l} w_{1}\left(t\right)=\lambda_{t-1}^{-1}F\left(t-1\right)w\left(t-1\right)\\ w_{j}\left(t\right)=\frac{U\left(t-1\right)U\left(t-2\right)\ldots U\left(t-j+1\right)F\left(t-j\right)w\left(t-j\right)}{\prod_{m=t-j}^{t-1}\lambda_{m}},\;j=2,3,\ldots \end{array}$$

because $\lambda_{t-1}^{-1}F\left(t-1\right)w\left(t-1\right)$ is the fraction of the population at t of those aged 1 (new recruits), the matrix $U\left(t-1\right)U\left(t-2\right)\ldots U\left(t-j+1\right)$ governs the survival and stage transitioning up to t and through age j≥2 of those who were new recruits at t−j+1. When they reach age j at t, the population has grown by a factor $\lambda_{t-1}\lambda_{t}\ldots \lambda_{t-j+1}$ since their first census.

When t<j, we should consider individuals that were already present in the initial population and are still alive at t. However, we do not really require an initial age distribution within the stages to compute $\partial \ln\lambda_{s}/\partial \theta_{j}$. Recall the weak ergodic theorem in demography: two populations experiencing the same sequence of projection matrices converge to the same time‐varying stage distribution even if they have different initial stage distributions (Cohen, 1979). A corollary of weak ergodicity is that the two populations also converge to the same age distribution even if their initial age distributions are different. In the long run, all initial individuals are dead and do not count directly anymore toward the age distribution. Moreover, the shared sequence of projection matrices and the convergence to a common stage distribution $w\left(t\right)$ imply that both the production of new recruits $F\left(t\right)w\left(t\right)$ and their subsequent survival, via $U\left(t+1\right)F\left(t\right)w\left(t\right)$, $U\left(t+2\right)U\left(t+1\right)F\left(t\right)w\left(t\right),\ldots \;,$ converge to the same levels in the two populations. The age distribution of a population is determined by the inflow of new individuals and their subsequent survival (Arthur, 1982). Hence, we expect the two populations to converge to the same time‐varying age distribution. Appendix B reports a numerical example of this convergence.

We can then estimate $\partial \ln\lambda_{s}/\partial \theta_{j}$ from long stochastic simulations starting from arbitrary age and stage distributions. Even more conveniently, we can outright discard a sufficient number of initial iterations of the simulated process so that any influence of the initial age distribution is negligible. Truncation of simulation results is already customary in the sensitivity analysis of $\ln\lambda_{s}$, for example, in Tuljapurkar et al. (2003), to minimize transient effects due to the initial stage distribution.

When the environment is constant and the population demographically stable, the recursion for $w_{j}$ in Equation 11 is

(16) $$w_{j+1}=\lambda^{-1}{\mathrm{Uw}}_{j},\;\text{with}\;w_{1}=\lambda^{-1}\mathrm{Fw}$$

as Cochran and Ellner (1992) showed, where w is the stable stage distribution.

## 3 APPLICATION 1—AGE‐SPECIFIC SELECTION, STAGES AND SENESCENCE

Hamilton (1966) kick‐started age‐specific sensitivity analysis of population growth. Equating lnλ with fitness, he proved that, in age‐classified stable populations, selection against mortality always declines with adult age, and so does selection on fecundity under mild assumptions (e.g., the population is not shrinking, λ≥1, and mortality is never zero). His results are key to understand the evolution of senescence, an age‐related decline in biological functioning (Baudisch, 2005; Caswell & Shyu, 2017; Charlesworth, 1994; Giaimo & Traulsen, 2022b; Partridge & Barton, 1993). However, Hamilton's results are based on an age‐classified model. To understand the stage‐classified case, Caswell and Salguero‐Gómez (2013) studied selection against mortality and on fecundity within stages for a s×s stage‐classified matrix model A with dominant eigenvalue λ by constructing from this matrix an age × stage matrix model comprising ω age classes. In the stage‐classified case, the selection force against mortality $\mu_{k,j}$ at age j within stage k is the sensitivity $\varphi \left(\mu_{k,j}\right)$ of lnλ to a proportional change of the same magnitude in survival (possibly accompanied by stage transitioning) for all individuals aged j in stage k in the population. This amounts to a proportional change of all entries in column k of $U_{j}=U$, where $U_{j}$ is the transition matrix for individuals aged j. In the stage‐classified case, such matrix is equal to the age‐independent transition matrix U. The selection force on fecundity $m_{k,j}$ at age j within a reproductive stage k is the sensitivity $\varphi \left(m_{k,j}\right)$ of lnλ to an additive change of the same magnitude in the fecundities of all individuals aged j in stage k in the population. This amounts to an additive change of the same magnitude in all positive entries of column k of $F_{j}=F$, where $F_{j}$ is the fecundity matrix for individuals aged j. In the stage‐classified case, such matrix is equal to the age‐independent transition matrix F. The stage‐independent quantities $\varphi \left(\mu_{j}\right)$ and $\varphi \left(m_{j}\right)$ are the sum of $\varphi \left(\mu_{k,j}\right)$ and the sum of $\varphi \left(m_{k,j}\right)$, respectively, over all stages k=1,…,s. The quantity $\varphi \left(\mu_{j}\right)$ is the selection force against mortality $\mu_{j}$ for all individuals aged j in the population regardless of their stage. The quantity $\varphi \left(m_{j}\right)$ is the selection force on fecundity $m_{j}$ for all individuals aged j independently of their stage. Let $\varphi_{\mu }\left(j\right)=\left(\varphi \left(\mu_{1,j}\right),\varphi \left(\mu_{2,j}\right),\ldots ,\varphi \left(\mu_{s,j}\right)\right)$ and $\varphi_{m}\left(j\right)=\left(\varphi \left(m_{1,j}\right),\varphi \left(m_{2,j}\right),\ldots ,\varphi \left(m_{s,j}\right)\right)$. Caswell and Salguero‐Gómez (2013, Eqs. 30‐1) proposed the following formulas for these quantities:
(17)

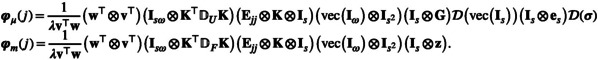

In Equation 17, ⊗ is the Kronecker product, $I_{n}$ is the n×n identity matrix, K is the sω×sω vec‐permutation matrix—see box 2 of Caswell et al. (2018) for more details on this matrix—, $D_{U}$ is a block diagonal matrix where each block is a ω×ω matrix with 1s in the whole subdiagonal and in the $\left(\omega ,\omega \right)$‐entry and zeros everywhere else, $D_{F}$ is a block diagonal matrix where each block is a ω×ω matrix with 1s in the first row and zeros everywhere else, $E_{\mathrm{jj}}$ is a matrix with a 1 in the $\left(j,j\right)$‐entry and zeros everywhere else, the $\mathrm{vec}\left(•\right)$ operator applies to a matrix and returns the columns of this stacked one on top of the other, G is the matrix U after normalizing each column so that its components add up to 1, $e_{s}$ is a s×1 vector of 1s, D is the operator that from a vector argument returns a diagonal matrix, σ is the vector of column sums of U and z is a s×1 vector where component i is 0 if row i of F is zero and 1 otherwise. Caswell and Salguero‐Gómez (2013) give more details on these expressions. They also noted that “[w]hile these expressions are impressive at first, they are easily evaluated in matrix‐oriented languages such as MATLAB” (p. 594). Using Equation 17, they numerically explored the trajectories of selective forces over age within stages in a sample of several plant species and observed that within‐stage selection can be contra‐senescent, that is, increasing over some ages, and not only pro‐senescent, that is, decreasing with age.

However, the remarkable observation by Caswell and Salguero‐Gómez (2013) of contra‐senescent selection has remained unexplained, as it is unclear what the determinants of contra‐senescent selection may be and when we should expect contra‐ or pro‐senescent selection. One way to get more insight would be to attack Equation 17 analytically. But we preferred to avoid facing the impressive expressions therein and we looked for an alternative approach based on our method. First of all, recall that the selection force against mortality $\mu_{j}$ at age j is the sensitivity of lnλ to a proportional change of the same magnitude in survival for all individuals aged j in the population regardless of their stage. Thus, we set a perturbed matrix as $A_{j}=F_{j}+\left(1+\theta_{j}\right)U_{j}$, where $U_{j}$ is the transition matrix for age class j. This matrix is identical to the overall transition matrix U in the absence of perturbations ($\theta_{j}=0$). From Equation 11, we then get

(18) $$\varphi \left(\mu_{j}\right)=\frac{\partial \ln\lambda }{\partial \theta_{j}}=\frac{v^{Τ}{\mathrm{Uw}}_{j}}{\lambda v^{Τ}w}.$$

Similarly, recall that the selection force on fecundity $m_{j}$ at age j is the sensitivity $\varphi \left(m_{j}\right)$ of lnλ to an additive increase of the same magnitude in the fecundities of all individuals aged j independently of their stage. Thus, we set a perturbed matrix as $A_{j}=U_{j}+F_{j}+\theta_{j}\mathrm{sgn}\left(F_{j}\right)$, where $F_{j}$ is the fecundity matrix for age class j, which is identical to the overall fecundity matrix F in the absence of perturbations ($\theta_{j}=0$), while $\mathrm{sgn}\left(F_{j}\right)$ is the matrix obtained by applying the signum function to $F_{j}$ entry‐wise, which returns a matrix with 1 in the $\left(i,j\right)$‐entry whenever the corresponding entry of $F_{j}$ is positive and zeros everywhere else (Section A.8 notes that alternative perturbation patterns are possible). From Equation 11, we then get

(19) $$\varphi \left(m_{j}\right)=\frac{\partial \ln\lambda }{\partial \theta_{j}}=\frac{v^{Τ}\mathrm{sgn}\left(F\right)w_{j}}{\lambda v^{Τ}w}.$$

Using Equations 18 and 19, we were able to derive analytic results that the present authors were not able to derive directly from Equation 17. To start putting our expressions at work, we employed Equation 16 and noted that death eventually completely erodes any newborn cohort. Therefore, $w_{j}$ tends to the zero vector as j→∞, see Section A.5 in the Appendix for a proof. The selective forces in Equations 18 and 19 have the same asymptotic behavior,

(20) $$\begin{array}{ll} \lim_{j\to \infty }\;\varphi \left(\mu_{j}\right) & =0,\\ \lim_{j\to \infty }\;\varphi \left(m_{j}\right) & =0, \end{array}$$

that is, selection vanishes at very late age in stage‐classified populations. This is hardly surprising. But, to our knowledge, a mathematical derivation of this fact within the formalism of stage‐classified populations was apparently missing in the literature and it might be seen as evidence of the analytic power of our method.

Section A.6 in the Appendix shows another result that can be obtained directly from Equation 18,

(21) $$\varphi \left(\mu_{j}\right)>\varphi \left(\mu_{j+1}\right),\;\text{with}\;j\geq \alpha -1$$

where α is the earliest reproductive age. This expression indicates that the force of selection against mortality steadily declines with reproductive age. This exactly mirrors a key result by Hamilton (1966) for the age‐classified case. No equivalent of Equation 21 exists for fecundity. Hence, the asymptotic behavior of $\varphi \left(m_{j}\right)$ does not rule out that, differently from Hamilton's original finding, selection on fecundity can increase over some ages before eventually waning (more on this below).

After having revisited some results of Hamilton (1966) for the stage‐classified case, we use Equations 18 and 19 to better understand age‐specific selection within stages. Since $\varphi \left(\mu_{j}\right)$ and $\varphi \left(m_{j}\right)$ have limit zero as j→∞, $\varphi \left(\mu_{j,k}\right)$ and $\varphi \left(m_{j,k}\right)$ must have the same asymptotic behavior. Evolutionarily, this means that selection within each stage has an overall pro‐senescent pattern at late ages. As for contra‐senescent selection, Section A.9 of the Appendix shows that

(22) $$\varphi \left(\mu_{k,j}\right)=b_{k}w_{k,j}\;\text{and}\;\varphi \left(m_{k,j}\right)=c_{k}w_{k,j},$$

where $b_{k}$ and $c_{k}$ are positive constants that depend on stage and not on age, whereas $w_{k,j}$ is the stable population fraction of age j and in stage k. Hence, within a stage, the age‐trajectory of selection against mortality and the age‐trajectory of selection on fecundity are both proportional to the stable age distribution within that stage.

**FIGURE 1:**
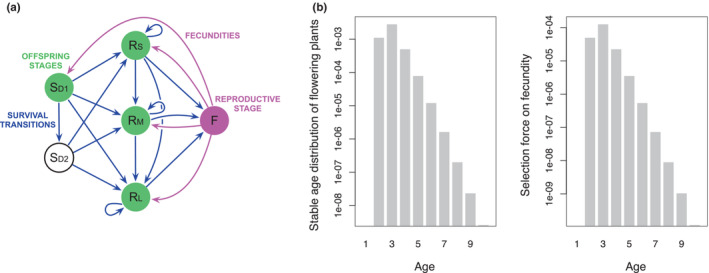
(a) Life cycle graph of *Dipsacus sylvestris*. Nodes represent stages. Blue arrows indicate survival transitions and correspond to entries of the U matrix. Magenta arrows indicate fecundities and correspond to entries of the F matrix. There is a single reproductive stage (magenta), Flowering plant, that can be accessed from any Rosette stage (small, medium and large). There are two Seed stages (dormant year 1 and dormant year 2). Offspring stages (green) are those where new recruits are first censused. (b) Stable age distribution of flowering plants and selection on their age‐specific fecundity in *Dipsacus*. Theoretical considerations (see main text) suggest that: age distribution and selection are proportional to one another, flowering plants can only be aged ≥2 and the age distribution initially increases and eventually decreases with age. Data for this analysis are from Caswell (2001, p. 60). For this model, stable population growth is λ=2.33 and individuals of every stage experience some nonzero mortality. The Supporting Information (Giaimo & Traulsen, 2022a) contains code to generate this panel.

At demographic stability, the overall age distribution monotonically decreases with age when the population is not going extinct because death progressively erodes any newborn cohort. The stable age distribution within a stage, instead, is not always monotonic, depends on details of the species life cycle and the shape of this distribution is not always obviously guessed (Boucher, 1997). However, some qualitative insights can be gained by reasoning upon the specific matrix model of one's interest. Take, for example, *Dipsacus sylvestris*, the wild teasel, as modeled by Caswell (2001, p. 60). Its life cycle is in Figure 1. It takes a minimum of two demographic projections to go from offspring stages, that is, stages where new recruits are found at their first census, to the unique reproductive stage (flowering plants). The reproductive stage is accessible from multiple stages where stasis, that is, permanence in the same stage upon projection, is possible. In turn, these stages can be accessed via multiple pathways. Hence, the age distribution within the reproductive stage should start increasing only at age 2 being progressively fueled by the arrival to that stage of individuals becoming reproductive at different ages. By proportionality between age distribution within a stage and selection on age‐specific fecundity within that stage, we expect selection on fecundity in *Dipsacus* to be initially contra‐senescent (Figure 1). Since there is a single reproductive stage, this also shows that stage‐independent selection on fecundity in this species can increase with adult age, in contrast to Hamilton's original finding.

**FIGURE 2:**
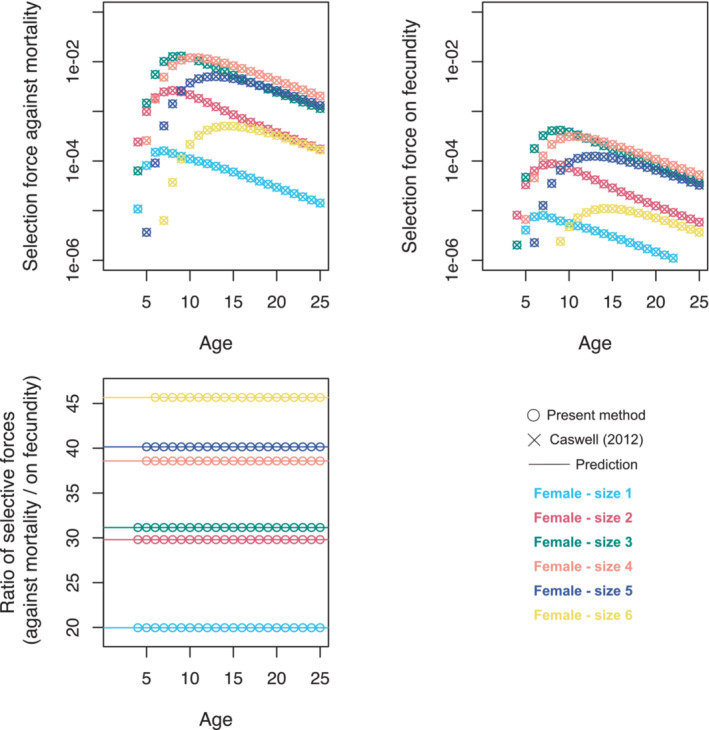
Age‐specific selective forces within females stages of *Arisaema serratum*. These forces are computed using the method proposed here (circles) and a previous method (diagonal crosses) by Caswell (2012) that Caswell and Salguero‐Gómez (2013, their fig. 3) employed to compute the same quantities. The methods are in perfect agreement. The distinctive analytical power of the proposed method, however, allows us to make a step further and predict (horizontal lines) from Equation 22 the existence of (and not only the theoretical possibility of visualizing) an age‐independent ratio between the force of selection against mortality and the force of selection on fecundity at each age within a single stage. The Supporting Information (Giaimo & Traulsen, 2022a) contains code to generate this figure.

Another key insight of Equation 22 is that, within a reproductive stage, selection against mortality and selection on fecundity are proportional to one another. In fact, Equation 22 immediately leads to

(23) $$\begin{array}{l} \frac{\varphi \left(\mu_{k,j}\right)}{\varphi \left(m_{k,j}\right)}=\frac{b_{k}}{c_{k}}, \end{array}$$

where $b_{k}/c_{k}$ is a quantity that depends on stage (k), yet not on age (j). This means that, within a stage, the magnitude of the ratio of selection against age‐specific mortality to selection on age‐specific fecundity remains constant independently of age. An illustration of this fact is in Figure 2, where we quantified selection within female stages of *Arisaema serratum* (Thunb.) Schott (Araceae), a perennial herb, as modeled by Kinoshita (1987). We also quantified selection with the method of Caswell (2012), which Caswell and Salguero‐Gómez (2013, Eqs. 30‐1) applied to the same dataset. The exact match with our results serves as a validation of our method. It should be noted that, through Equation 17 by Caswell and Salguero‐Gómez (2013), one can also plot the ratio of these two selective forces (Caswell, personal communication). However, as with the other results above, we were not able to prove as directly from Equation 17 the age independence of this ratio.

It should also be remarked that our findings about age‐specific selective forces apply to the case of models only classified by stage. To derive these results, we have leveraged the fact that the same transition matrix and the same fecundity matrix apply to each age class in these models. In age × stage matrix models, which we do not study here, the transition matrix and the fecundity matrix typically differ between age classes so that our results do not obviously generalize to these models.

## 4 APPLICATION 2—AGE‐SPECIFIC ELASTICITIES

Elasticities are proportional sensitivities (Caswell, 2001). They are much used quantities in ecological analysis, for example (Coutts et al., 2016; Csergő et al., 2017; Kayal et al., 2018; Koons et al., 2016; Struckman et al., 2019; Tredennick et al., 2018). Here, we show how to decompose the elasticities of population growth for a stage‐classified matrix model into their age‐specific components. We shall concentrate on elasticities of population growth to entries of the matrix model, and not to lower level parameters.

We are interested in the elasticity of λ to a subset of the entries of the stage‐classified matrix model A. Let $A_{0}$ be the matrix obtained from A by zeroing the entries that are not of our interest. Creating a perturbed matrix $A+\theta A_{0}$ with the unperturbed case given by θ=0 and using Equation 2, the relevant elasticity is

(24) $$\varepsilon \left(\lambda ,A_{0}\right)=\frac{v^{Τ}A_{0}w}{\lambda v^{Τ}w}.$$

Using Equation 11, making this elasticity age‐specific is very simple

(25) $$\varepsilon_{j}\left(\lambda ,A_{0}\right)=\frac{v^{Τ}A_{0}w_{j}}{\lambda v^{Τ}w},$$

This is the sensitivity of lnλ to the entries of A that are of our interest when these are proportionally perturbed only for individuals of age j.

By a similar reasoning as before applied to Equation 6, the age‐independent elasticity of the stochastic growth rate to a subset of the entries of the matrix model is

(26) $$\varepsilon \left(\lambda_{s},A_{0}\right)=\lim_{L\to \infty }\frac{1}{L}\sum_{t=0}^{L-1}\frac{v^{Τ}\left(t+1\right)A_{0}\left(t\right)w\left(t\right)}{\lambda_{t}v^{Τ}\left(t+1\right)w\left(t+1\right)}.$$

It has proven important for some analyses to decompose this stochastic elasticity in different ways. Tuljapurkar et al. (2003) and Haridas and Tuljapurkar (2005) distinguished the elasticity of $\lambda_{s}$ to the averages of the relevant entries of the matrix model

(27) $$\varepsilon \left(\lambda_{s},{\bar{A}}_{0}\right)=\lim_{L\to \infty }\frac{1}{L}\sum_{t=0}^{L-1}\frac{v^{Τ}\left(t+1\right){\bar{A}}_{0}w\left(t\right)}{\lambda_{t}v^{Τ}\left(t+1\right)w\left(t+1\right)},$$

from the elasticity of $\lambda_{s}$ to the standard deviations of these entries

(28) $$\varepsilon \left(\lambda_{s},A_{0}-{\bar{A}}_{0}\right)=\lim_{L\to \infty }\frac{1}{L}\sum_{t=0}^{L-1}\frac{v^{Τ}\left(t+1\right)\left(A_{0}\left(t\right)-{\bar{A}}_{0}\right)w\left(t\right)}{\lambda_{t}v^{Τ}\left(t+1\right)w\left(t+1\right)},$$

where ${\bar{A}}_{0}$ is the long‐run average of $A_{0}\left(t\right)$. Note that $\varepsilon \left(\lambda_{s},{\bar{A}}_{0}\right)+\varepsilon \left(\lambda_{s},A_{0}-{\bar{A}}_{0}\right)=\varepsilon \left(\lambda_{s},A_{0}\right)$, see Tuljapurkar et al. (2003). Hence, the elasticity in Equation 26 can be seen as an elasticity to both the average and the standard deviation of the entries of interest.

The other decomposition of Equation 26 that we will consider here is into environment‐specific elasticities. Let ξ be a subset of the environmental states and let $1_{\xi }\left(t\right)$ be an indicator function that takes value 1 at t when the environment is in a state in ξ and value 0 otherwise. The elasticity

(29) $$\varepsilon \left(\lambda_{s},1_{\xi }A_{0}\right)=\lim_{L\to \infty }\frac{1}{L}\sum_{t=0}^{L-1}\frac{v^{Τ}\left(t+1\right)1_{\xi }\left(t\right)A_{0}\left(t\right)w\left(t\right)}{\lambda_{t}v^{Τ}\left(t+1\right)w\left(t+1\right)},$$

is the sensitivity of $\ln\lambda_{s}$ to a proportional perturbation of the entries of our interest in the matrix model only when the environment is in a state in ξ (Åberg et al., 2009; Caswell, 2005; Horvitz et al., 2005). When all environmental states are in ξ, the elasticity in Equation 29 is equal to the environment‐independent elasticity in Equation 26. When ξ contains a single environmental state, we refer to the resulting elasticity as the elasticity of $\lambda_{s}$ to that environment. However, note that this name should not suggest in any way a change in the transition probabilities of the (assumed constant) environmental Markov chain. For perturbations of this chain, see Steinsaltz et al. (2011).

Making elasticities of the stochastic growth rate age‐dependent is just as easy as it was for the stable growth rate. Using Equation 12, all it takes is to replace $w\left(t\right)$ in the numerator of Equations (26), (27), (28), (29) with $w_{j}\left(t\right)$,

(30a) $$\varepsilon_{j}\left(\lambda_{s},A_{0}\right)=\lim_{L\to \infty }\frac{1}{L}\sum_{t=0}^{L-1}\frac{v^{Τ}\left(t+1\right)A_{0}\left(t\right)w_{j}\left(t\right)}{\lambda_{t}v^{Τ}\left(t+1\right)w\left(t+1\right)}$$

(30b) $$\varepsilon_{j}\left(\lambda_{s},{\bar{A}}_{0}\right)=\lim_{L\to \infty }\frac{1}{L}\sum_{t=0}^{L-1}\frac{v^{Τ}\left(t+1\right){\bar{A}}_{0}w_{j}\left(t\right)}{\lambda_{t}v^{Τ}\left(t+1\right)w\left(t+1\right)}$$

(30c) $$\varepsilon_{j}\left(\lambda_{s},A_{0}-{\bar{A}}_{0}\right)=\lim_{L\to \infty }\frac{1}{L}\sum_{t=0}^{L-1}\frac{v^{Τ}\left(t+1\right)\left(A_{0}\left(t\right)-{\bar{A}}_{0}\right)w_{j}\left(t\right)}{\lambda_{t}v^{Τ}\left(t+1\right)w\left(t+1\right)}$$

(30d) $$\varepsilon_{j}\left(\lambda_{s},1_{\xi }A_{0}\right)=\lim_{L\to \infty }\frac{1}{L}\sum_{t=0}^{L-1}\frac{v^{Τ}\left(t+1\right)1_{\xi }\left(t\right)A_{0}\left(t\right)w_{j}\left(t\right)}{\lambda_{t}v^{Τ}\left(t+1\right)w\left(t+1\right)}.$$

These elasticities have the same meaning as their age‐independent counterparts except that they refer to perturbations that only affect individuals of age j.

Finally, we consider elasticities of transient growth. From Equations 7 and 8, the elasticity of $\lambda_{t}$ to a subset of the entries of A is

(31) $$\varepsilon \left(\lambda_{t},A_{0}\right)=\frac{e^{Τ}}{N\left(t+1\right)}\frac{\partial n\left(t+1\right)}{\partial \theta }-\frac{e^{Τ}}{N\left(t\right)}\frac{\partial n\left(t\right)}{\partial \theta },\;\text{with}\;\frac{\partial n\left(t+1\right)}{\partial \theta }=A_{0}n\left(t\right)+A\left(t\right)\frac{\partial n\left(t\right)}{\partial \theta }.$$

From Equations 13 and 14, the age‐specific version of this quantity simply is

(32) $$\varepsilon_{j}\left(\lambda_{t},A_{0}\right)=\frac{e^{Τ}}{N\left(t+1\right)}\frac{\partial n\left(t+1\right)}{\partial \theta_{j}}-\frac{e^{Τ}}{N\left(t\right)}\frac{\partial n\left(t\right)}{\partial \theta_{j}},\;\text{with}\;\frac{\partial n\left(t+1\right)}{\partial \theta_{j}}=A_{0}n_{j}\left(t\right)+A\left(t\right)\frac{\partial n\left(t\right)}{\partial \theta_{j}}.$$

Since the sum of $w_{j}\left(t\right)$ over all ages j is $w\left(t\right)$, all age‐specific elasticities we have considered above decompose the corresponding age‐independent elasticities

(33) $$\sum_{j=1}^{\infty }\varepsilon_{j}=\varepsilon .$$

Assuming, as it seems reasonable, that even under fluctuating environment, death stills erodes to the end any initial newborn cohort so that $w_{j}\left(t\right)\to 0$ as j→∞, then Equations 30 and 32 imply that $\varepsilon_{j}\to 0$ as j→∞. Hence, we can approximate an age‐independent elasticity ε by adding a sufficient number of its age‐specific components $\varepsilon_{j}$.

To put age‐specific stochastic elasticities at work, we took demographic data about one of the populations of *Anthyllis vulneraria*, the common kidney vetch, analyzed by Davison et al. (2010). In their model, there are three projection matrices corresponding to three environmental states assumed independent and identically distributed. Four stages were distinguished: seedling, vegetative adult, small flowering adult, and large flowering adult. Projection matrices were of the form

(34) $$A\left(t\right)=\left(\begin{array}{cccc} 0 & 0 & F_{1,3}\left(t\right) & F_{1,4}\left(t\right)\\ G_{2,1}\left(t\right) & {\mathrm{St}}_{2,2}\left(t\right) & R_{2,3}\left(t\right) & R_{2,4}\left(t\right)\\ G_{3,1}\left(t\right) & G_{3,2}\left(t\right) & {\mathrm{St}}_{3,3}\left(t\right) & R_{3,4}\left(t\right)\\ G_{4,1}\left(t\right) & G_{4,2}\left(t\right) & G_{4,3}\left(t\right) & {\mathrm{St}}_{4,4}\left(t\right) \end{array}\right).$$

**FIGURE 3:**
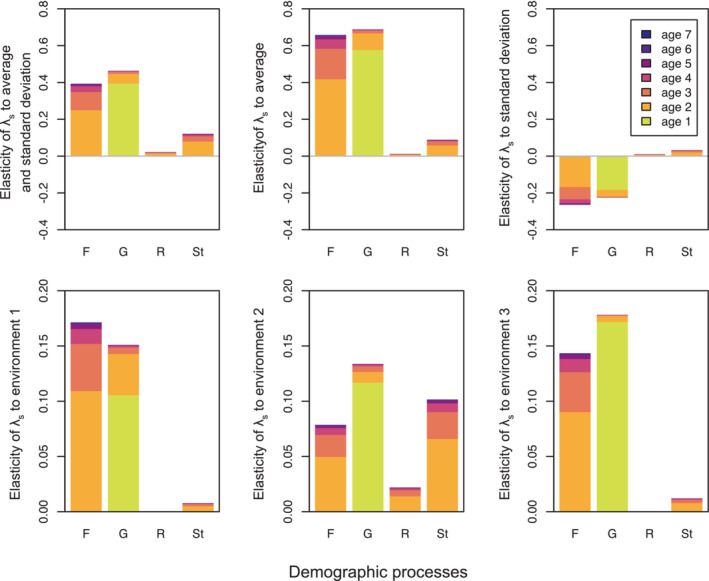
Age‐specific elasticities of stochastic growth $\lambda_{s}$. We initiated a population with random stage distribution. We demographically updated the populations for 70,000 time‐steps using a randomly generated sequence of projection matrices. We computed elasticities of $\lambda_{s}$ to different demographic processes: fecundity (F), individual growth (G), retrogression (R), and stasis (St). Age‐specific elasticities were computed for each case using Equation 30. Only age‐specific elasticities up to age 7 are reported here, as elasticities for later ages contributed only minimally to the overall age‐independent elasticity for this dataset. In computing elasticities, we removed the first and last 10,000 steps to minimize transient effects. The analysis is based on demographic data for population E of kidney vetch in Davison et al. (2010). The Supporting Information (Giaimo & Traulsen, 2022a) contains code to generate this figure.

Entries in this matrix are grouped on the basis of the general demographic process they contribute to: fecundity (F), individual growth (G), retrogression (R), and stasis (St). To obtain the elasticity of $\lambda_{s}$ to one of these processes, we set $A_{0}\left(t\right)$ by zeroing the entries in Equation 34 that do not pertain to this process. Figure 3 reports stochastic elasticities to each demographic process. Elasticities are represented as sums of age‐specific components. Figure 3 makes visible how, overall, individual growth at age 1 is the age‐specific demographic process to which $\lambda_{s}$ is the most elastic.

**FIGURE 4:**
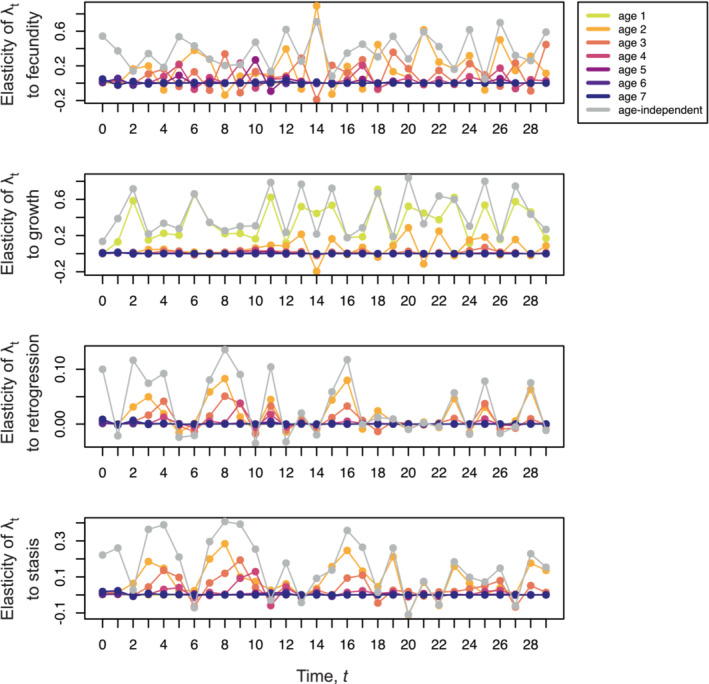
Age‐specific elasticities of transient growth $\lambda_{t}$. We initiated a population with uniform stage distribution and random age distribution with maximum initial age of 30. We demographically updated the populations for 30 time‐steps using a randomly generated sequence of projection matrices. We computed elasticities of $\lambda_{t}$ to different demographic processes: fecundity (F), individual growth (G), retrogression (R), and stasis (St). Age‐specific elasticities were computed for each case using Equation 32 with initial vectors $\partial n\left(0\right)/\partial \theta$ set equal to the zero vector. Only age‐specific elasticities up to age 7 are reported here, as elasticities for later ages contributed only minimally to the overall age‐independent elasticity for t≥6 in these simulations. Note that elasticities are on different scales. The analysis is based on demographic data for population E of kidney vetch in Davison et al. (2010). The Supporting Information (Giaimo & Traulsen, 2022a) contains code to generate this figure.

Using the same dataset, we computed age‐specific elasticities of transient growth to each general demographic process (Figure 4). Age‐independent elasticities of transient growth can take different signs at different time points (see elasticity to stasis, Figure 4). Our analysis shows that the elasticity of transient growth at a single time point can have age‐specific components of different signs. For example, looking at time t=14 in Figure 4, the elasticity to fecundity at age 2 is positive, while the elasticity to fecundity at age 3 is negative.

## 5 SUMMARY

The growth rate of a population is a quantity of primary interest in ecology and evolution. A well‐established machinery rooted in matrix models exists to compute the sensitivity of population growth to any underlying demographic variable (Caswell, 2001, 2005, 2007, 2019; Caswell et al., 2018; Horvitz et al., 2005; Tuljapurkar et al., 2003). Here, we have proposed a method that, by only minimally modifying some results of such machinery, can make sensitivity analysis age‐specific. The modification essentially consists in replacing the overall stage distribution that appears in sensitivity formulas for stage‐classified populations with the age‐specific stage distribution. The latter is easily computed by distinguishing transition/survival events from reproductive events in the matrix model. The method may be useful to those who are interested in analyzing along the “age” dimension the already existing matrix models that are classified solely by stage.

The proposed method applies to stable population growth, the stochastic growth rate and transient growth. In the age‐specific sensitivity analysis of stable growth for populations solely classified by stage, our method offers a possible alternative to the established method that requires for this analysis the separate construction of an age × stage matrix model (Caswell, 2012; Caswell et al., 2018). This latter method led to observe contra‐ and pro‐senescent selection within life stages (Caswell & Salguero‐Gómez, 2013). We regard as an advantage of our method its ability to explain these observations by proving the existence of a proportionality between age‐specific selective forces within a stage and the age distribution within that stage. In the case of stochastic population growth, to our knowledge there is no other method that has been applied explicitly yet for the age‐specific sensitivity analysis of matrix models only classified by stage.

More generally, our method contributes to studies in ecology and evolution by decomposing usual quantities in the sensitivity analysis of population growth, like elasticities, into their age‐specific components. Thus, the proposed method makes sensitivity analysis more fine‐grained.

## AUTHOR CONTRIBUTIONS

**Stefano Giaimo:** Conceptualization (lead); data curation (lead); formal analysis (lead); investigation (lead); methodology (lead); project administration (lead); software (lead); validation (lead); visualization (lead); writing – original draft (lead); writing – review and editing (lead). **Arne Traulsen:** Funding acquisition (lead); supervision (supporting); validation (supporting); writing – review and editing (supporting).

## CONFLICT OF INTEREST

The authors have no conflicts of interest to declare.

### OPEN RESEARCH BADGES

This article has earned Open Data and Open Materials badges. Data and materials are available at https://doi.org/10.5061/dryad.6m905qg3m.

## Figures and Tables

**FIGURE B1:**
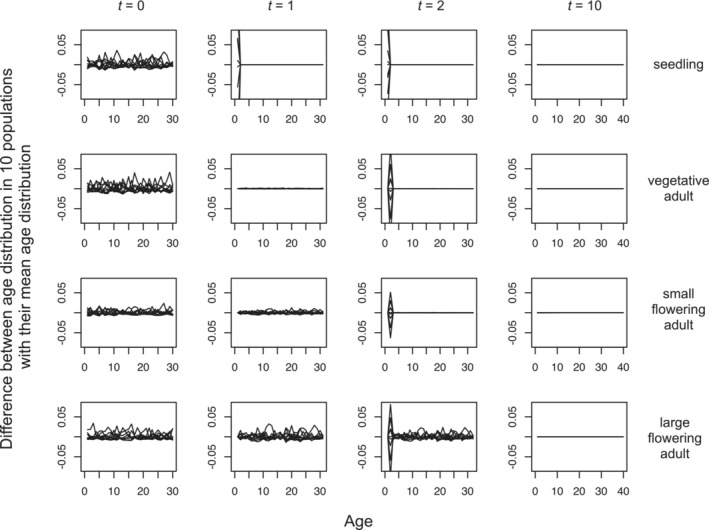
Convergence to a common age distribution. We used demographic data for population C of kidney vetch in Davison et al. (2010). We initiated ten populations. To each, we assigned a random age and stage distribution with maximum age 30. We updated the populations for several time steps by subjecting the populations to the same randomly generated sequence of projection matrices. Here, we report for different time points (columns) and within each stage (row), the differences between the age distribution of each population and the arithmetic mean of the ten age distributions. Different lines correspond to different populations. As time increases, differences vanish and lines overlap so that, eventually, only a single horizontal line at level 0 is visible. The Supporting Information (Giaimo & Traulsen, 2022a) contains code to generate this figure.

## Data Availability

Code and data that support the findings of this study are openly available in Dryad at https://doi.org/10.5061/dryad.6m905qg3m.

## APPENDIX A MATHEMATICAL DERIVATIONS

### A.1 Derivation of Equation 11

We start from the eigenvector equation that relates the stable stage distribution w (with components that add up to 1), the stable growth rate λ and the projection matrix A of the population:

(A1)
λw=Aw.

We rewrite the right hand side of Equation A1 as a series

(A2)
$$\mathrm{Aw}=\sum_{k=1}^{\infty }A_{k}w_{k},$$

where the component i of $w_{k}$ is the stable population fraction that is both in stage i and of age k. Age 1 is assigned to new recruits at their first census. Age is updated at demographic projection so that an individual of age j at t is of age j+1 at t+1. Thus, w can be thought of as the row sums of an array with as many rows as stages and an infinite number of columns each representing one age. The sum over all entries of the array is 1. The age index in Equation A2 runs to infinity. To set a maximum age ω, it is sufficient to let all vectors $w_{j}$ with j>ω be equal to the zero vector. In Equation A2, $A_{k}=A$ for all k=1,2,… so that $A_{k}w_{k}$ demographically projects individuals aged k over one time step.

In this scheme, individuals of each age are all subject to the same demographic rates. But suppose there is a parameter $x_{j}$ on which the demographic rates of individuals of age j in the population depend. The parameter currently takes value $x_{j}^{*}$. Since the demography of the population depends on $x_{j}$, both the stable stage distribution w and the stable growth rate λ are functions of this parameter. As for demographic projection, only $A_{j}$ is a function of $x_{j}$, while all other matrices $A_{k}$ with k≠j in Equation A2 are not, as these matrices contain the demographic rates to project individuals of age different from j. However, note that there presently is no difference between these projection matrices so that $A_{j}\left(x_{j}^{*}\right)=A_{k}$ with k≠j. To express the dependencies of λ, w, and $A_{j}$ from $x_{j}$ we can rewrite Equation A1 using Equation A2

(A3)
$$\lambda \left(x_{j}\right)w\left(x_{j}\right)=\sum_{\begin{array}{c} k=1\\ k\neq j \end{array}}^{\infty }A_{k}w_{k}\left(x_{j}\right)+A_{j}\left(x_{j}\right)w_{j}\left(x_{j}\right).$$

Note that, when $x_{j}=x_{j}^{*}$, the right hand side of Equation A3 converges to Aw. We assume that $\sum_{k=1}^{\infty }\partial w_{j}/\partial x_{j}$ uniformly converges on some interval containing $x_{j}^{*}$. Let us differentiate Equation A3 with respect to $x_{j}$

(A4)
$$\begin{array}{ll} \frac{\partial \lambda \left(x_{j}\right)}{\partial x_{j}}w\left(x_{j}\right)+\lambda \left(x_{j}\right)\frac{\partial w\left(x_{j}\right)}{\partial x_{j}} & =\sum_{\begin{array}{c} k=1\\ k\neq j \end{array}}^{\infty }A_{k}\frac{\partial w_{k}\left(x_{j}\right)}{\partial x_{j}}+\frac{\partial A_{j}\left(x_{j}\right)}{\partial x_{j}}w_{j}\left(x_{j}\right)+A_{j}\left(x_{j}\right)\frac{\partial w_{j}\left(x_{j}\right)}{\partial x_{j}}. \end{array}.$$

Multiplying v, the reproductive value vector, on the left of Equation A4,

(A5)
$$\begin{array}{l} \frac{\partial \lambda \left(x_{j}\right)}{\partial x_{j}}v^{Τ}\left(x_{j}\right)w\left(x_{j}\right)+\lambda \left(x_{j}\right)v^{Τ}\left(x_{j}\right)\frac{\partial w\left(x_{j}\right)}{\partial x_{j}}=v^{Τ}\left(x_{j}\right)\sum_{\begin{array}{c} k=1\\ k\neq j \end{array}}^{\infty }A_{k}\frac{\partial w_{k}\left(x_{j}\right)}{\partial x_{j}}\\ \;+v^{Τ}\left(x_{j}\right)\frac{\partial A_{j}\left(x_{j}\right)}{\partial x_{j}}w_{j}\left(x_{j}\right)+v^{Τ}\left(x_{j}\right)A_{j}\left(x_{j}\right)\frac{\partial w_{j}\left(x_{j}\right)}{\partial x_{j}}. \end{array}$$

Evaluating Equation A5 at $x_{j}=x_{j}^{*}$,

(A6)
$$\begin{array}{ll} {\left.\frac{\partial \lambda \left(x_{j}\right)}{\partial x_{j}}\right|}_{{}_{x_{j}=x_{j}^{*}}}v^{Τ}\left(x_{j}^{*}\right)w\left(x_{j}^{*}\right)+\lambda \left(x_{j}^{*}\right)v^{Τ}\left(x_{j}^{*}\right){\left.\frac{\partial w\left(x_{j}\right)}{\partial x_{j}}\right|}_{x_{j}=x_{j}^{*}}= & v^{Τ}\left(x_{j}^{*}\right)\sum_{\begin{array}{c} k=1\\ k\neq j \end{array}}^{\infty }A_{k}{\left.\frac{\partial w_{k}\left(x_{j}\right)}{\partial x_{j}}\right|}_{x_{j}=x_{j}^{*}}\\ & +v^{Τ}\left(x_{j}^{*}\right){\left.\frac{\partial A_{j}\left(x_{j}\right)}{\partial x_{j}}\right|}_{x_{j}=x_{j}^{*}}w_{j}\left(x_{j}^{*}\right)\\ & +v^{Τ}\left(x_{j}^{*}\right)A_{j}\left(x_{j}^{*}\right){\left.\frac{\partial w_{j}\left(x_{j}\right)}{\partial x_{j}}\right|}_{x_{j}=x_{j}^{*}}. \end{array}$$

Using the fact that the reproductive value vector is the left eigenvector of the population projection matrix when $x_{j}=x_{j}^{*}$, we have that

(A7)
$$\lambda \left(x_{j}^{*}\right)v^{Τ}\left(x_{j}^{*}\right)=v^{Τ}\left(x_{j}^{*}\right)A_{j}\left(x_{j}^{*}\right)=v^{Τ}\left(x_{j}^{*}\right)A_{k},\;k\neq j$$

Using Equation A7 into Equation A6, simplifying and rearranging leads to

(A8)
$${\left.\frac{\partial \lambda }{\partial x_{j}}\right|}_{x_{j}=x_{j}^{*}}=\frac{{\left.v^{Τ}\left(x_{j}^{*}\right)\frac{\partial A_{j}\left(x_{j}\right)}{\partial x_{j}}\right|}_{x_{j}=x_{j}^{*}}w_{j}\left(x_{j}^{*}\right)}{v^{Τ}\left(x_{j}^{*}\right)w_{j}\left(x_{j}^{*}\right)},$$

dividing this equation by $\lambda \left(x_{j}^{*}\right)$ yields

(A9)
$${\left.\frac{\partial \ln\lambda }{\partial x_{j}}\right|}_{x_{j}=x_{j}^{*}}=\frac{{\left.v^{Τ}\left(x_{j}^{*}\right)\frac{\partial A_{j}\left(x_{j}\right)}{\partial x_{j}}\right|}_{x_{j}=x_{j}^{*}}w_{j}\left(x_{j}^{*}\right)}{\lambda \left(x_{j}^{*}\right)v^{Τ}\left(x_{j}^{*}\right)w_{j}\left(x_{j}^{*}\right)},$$

which corresponds to Equation 11 in the main text.

### A.2 Equivalence with Caswell (2012)

Here, we show that the approach of Caswell (2012) to the age‐specific sensitivity analysis of stable population growth for stage‐classified models and our approach are essentially equivalent. Given a q×q, time‐independent stage‐classified model A, Caswell (2012) suggests to decompose it into a transition matrix $U=\left(u_{i,j}\right)$ and a fecundity matrix $F=\left(f_{i,j}\right)$, where $u_{i,j}$ is the probability that an individual in stage j at t is alive and observed in stage i at t+1 and $f_{i,j}$ is the number of new recruits in the population observed in stage i at t+1 per individual in stage j at t, so that A=U+F. Then, fixing a maximum number ω of age classes, one defines the age‐specific matrices $U_{i}=U$ and $F_{i}=F$ for i=1,2,…,ω, where $U_{i}+F_{i}=A_{i}$ with $A_{i}$ defined as in Section 2.2 of the main text. Using these matrices and a construction approach of extremely high generality, Caswell (2012) builds the age × stage matrix $\tilde{A}$ in blocks. It would seem that Caswell's construction leads to:

(A10)
$$\tilde{A}=\left(\begin{array}{ccccc} F_{1} & F_{2} & F_{3} & \ldots & F_{\omega }\\ U_{1} & & & & \\ & U_{2} & & & \\ & & \ddots & & \\ & & & U_{\omega -1} & U_{\omega } \end{array}\right).$$

This matrix updates a population state vector $\tilde{n}\left(t\right)$ of the form

(A11)
$$\tilde{n}\left(t\right)=\left(\begin{array}{c} {\tilde{n}}_{1}\left(t\right)\\ {\tilde{n}}_{2}\left(t\right)\\ \vdots \\ {\tilde{n}}_{\geq \omega }\left(t\right) \end{array}\right),$$

where ${\tilde{n}}_{k}\left(t\right)={\left({\tilde{n}}_{1,k}\left(t\right),{\tilde{n}}_{2,k}\left(t\right),\ldots ,{\tilde{n}}_{q,k}\left(t\right)\right)}^{Τ}$ with k=1,2,…,ω−1 is the vector containing the population abundance ${\tilde{n}}_{i,k}\left(t\right)$ in stage i and of age k at t, while the vector ${\tilde{n}}_{\geq \omega }\left(t\right)={\left({\tilde{n}}_{1,\geq \omega }\left(t\right),{\tilde{n}}_{2,\geq \omega }\left(t\right),\ldots ,{\tilde{n}}_{q,\geq \omega }\left(t\right)\right)}^{Τ}$ contains the population abundance ${\tilde{n}}_{i,\geq \omega }\left(t\right)$ in stage i and of age at least ω at t. Note, in particular, that the shape of $\tilde{A}$ implies that new recruits at t are all in ${\tilde{n}}_{1}\left(t\right)$ and that individuals in ${\tilde{n}}_{k}\left(t\right)$ who survive (and possibly transition of stage) over one time step are all in ${\tilde{n}}_{k+1}\left(t+1\right)$ for k=1,2,…,ω−2, those who are in ${\tilde{n}}_{\omega -1}\left(t\right)$ and survive over one time step are all in ${\tilde{n}}_{\geq \omega }\left(t+1\right)$ with the remaining part of those in ${\tilde{n}}_{\geq \omega }\left(t+1\right)$ being composed of those who are in ${\tilde{n}}_{\geq \omega }\left(t\right)$ and survive over one time step. This explains the sense in which the matrices $U_{i}$ and $F_{i}$ are age‐specific. Caswell (2012) then proposes to get age‐specific sensitivity information about the initial, stage‐classified matrix A by performing the analysis on the age × stage matrix $\tilde{A}$ instead. The assumption is that A and $\tilde{A}$ have the same dominant eigenvalue. As Caswell (2012, p. 409) says, since “the vital rates do not depend on age, the dominant eigenvalues of A and $\tilde{A}$ should be identical.” Caswell (2012, p. 413) also notes that, in general, $\tilde{A}$ may be reducible. “This means that one must ascertain that the eigenvalues and eigenvectors [of the age × stage model] under analysis correspond to initial conditions of interest” (Caswell, 2012, p. 413), because asymptotic dynamics may depend on initial conditions. However, Caswell (2012, pp. 413, 415–6) shows that it is both necessary and sufficient that the left eigenvector corresponding to the dominant eigenvalue of $\tilde{A}$ is positive component‐wise for population growth to be described by this eigenvalue regardless of initial conditions. Caswell (2012) appears to suggest that one should numerically check for the specific matrix model of one's interest whether the condition of positivity for the left dominant eigenvector of $\tilde{A}$ is satisfied.

Before showing equivalence with our approach, we first expand on Caswell's approach. We assume that A is irreducible. By irreducibility and nonnegativity, the matrix A has a dominant eigenvalue λ, which is real, positive, with geometric multiplicity of 1 and greater in magnitude than all other eigenvalues of A (Horn & Johnson, 2013, Theorem. 8.4.4). Let w and v be the unique (up to a constant scalar) right and left eigenvectors of A, respectively, corresponding to λ. By Perron–Frobenius theorem, these eigenvectors are both positive component‐wise (ibid.). Recall that the matrix $\tilde{A}$ is nonnegative and possibly reducible. Let us form the equations $xy=\tilde{A}y$ and $xz^{Τ}=z^{Τ}\tilde{A}$ with unknowns x (scalar), y and z (vectors). Recall Equation A16 from the main text,

(A12)
$$\left\{\begin{array}{l} w_{1}=\lambda^{-1}\mathrm{Fw}\\ w_{j+1}=\lambda^{-1}{\mathrm{Uw}}_{j}, \end{array}\right.$$

where w is the dominant right eigenvector of A, that is, the stable stage distribution, A=U+F and $w_{j}$ is the fraction of the stable stage distribution aged j so that $\sum_{i=1}^{\infty }w_{i}=w$. Define $w_{\geq \omega }=\sum_{i=\omega }^{\infty }w_{j}$ and observe that

(A13)
$$U\left(w_{\omega -1}+w_{\geq \omega }\right)=\lambda w_{\omega }+U\sum_{i=\omega }^{\infty }w_{i}=\lambda w_{\omega }+\sum_{i=\omega }^{\infty }{\mathrm{Uw}}_{i}=\lambda w_{\omega }+\sum_{i=\omega }^{\infty }\lambda w_{i+1}=\lambda \left(w_{\omega }+\sum_{i=\omega +1}^{\infty }w_{i}\right)=\lambda w_{\geq \omega }.$$

We can see that x=λ and $y={\left(w_{1},w_{2},\ldots ,w_{\geq \omega }\right)}^{Τ}$ are solutions of the equation $xy=\tilde{A}y$, because from Equations (A10), (A11), (A12), (A13), $U_{i}=U$ and $F_{i}=F$,

(A14)
$$\begin{array}{l} \tilde{A}\left(\begin{array}{c} w_{1}\\ w_{2}\\ w_{3}\\ \vdots \\ w_{\geq \omega } \end{array}\right)=\left(\begin{array}{c} F_{1}w_{1}+F_{2}w_{2}+...+F_{\omega }w_{\geq \omega }\\ U_{1}w_{1}\\ U_{2}w_{2}\\ \vdots \\ U_{\omega -1}w_{\omega -1}+U_{\omega }w_{\geq \omega } \end{array}\right)=\left(\begin{array}{c} F\left(w_{1}+w_{2}+...+w_{\geq \omega }\right)\\ {\mathrm{Uw}}_{1}\\ {\mathrm{Uw}}_{2}\\ \vdots \\ U\left(w_{\omega -1}+w_{\geq \omega }\right) \end{array}\right)=\lambda \left(\begin{array}{c} w_{1}\\ w_{2}\\ w_{3}\\ \vdots \\ w_{\geq \omega } \end{array}\right). \end{array}$$

Similarly, x=λ and $z={\left(v^{Τ},v^{Τ},\ldots ,v^{Τ}\right)}^{Τ}$ are solutions of the equation $\lambda z^{Τ}=z^{Τ}\tilde{A}$, because v is the left eigenvector corresponding to the eigenvalue λ of A=U+F (with $U=U_{i}$ and $F=F_{i}$) and, therefore, using Equation A10

(A15)
$$\begin{array}{ll} \left(v^{Τ},v^{Τ},\ldots ,v^{Τ}\right)\tilde{A} & =\left(v^{Τ}\left(F_{1}+U_{1}\right),v^{Τ}\left(F_{2}+U_{2}\right),\ldots ,v^{Τ}\left(F_{\omega }+U_{\omega }\right)\right)\\ & =\left(v^{Τ}A,v^{Τ}A,\ldots ,v^{Τ}A\right)\\ & =\lambda \left(v^{Τ},v^{Τ},\ldots ,v^{Τ}\right). \end{array}$$

Using these results and the fact that v is positive component‐wise, Theorem 8.3.4 in Horn and Johnson (2013) implies that λ is a dominant eigenvalue of $\tilde{A}$ and a corresponding left eigenvector is component‐wise positive. Letting then $I_{\mathrm{q\omega}}$ be the qω×qω identity matrix, we can see from Equation A10 that $\lambda I_{\mathrm{q\omega}}-\tilde{A}$ has at least some positive principal minor, for example, its $\left(q+1,q+1\right)$‐entry. Therefore, by Problem 8.3.P14 in Horn and Johnson (2013), the eigenvalue λ of $\tilde{A}$ has geometric multiplicity at most 1, that is, its left and right eigenvectors are unique. We can then strengthen the informal considerations and results of Caswell (2012) by concluding that the irreducibility of A and the construction of $\tilde{A}$ from it are sufficient both to identify the dominant eigenvalue λ of A with the dominant eigenvalue of $\tilde{A}$ and to guarantee that this eigenvalue describes population growth when the population is updated via $\tilde{A}$ independently of initial conditions, that is, if A is irreducible, there is no need to numerically check the positivity of the left dominant eigenvector of $\tilde{A}$, as this positivity is already guaranteed by the irreducibility of A.

We can now directly address the equivalence problem. Let us denote with $\tilde{w}$ and $\tilde{v}$, respectively, the right and left eigenvectors of $\tilde{A}$ and note that, using the above results about these eigenvectors,

(A16)
$${\tilde{v}}^{Τ}\tilde{w}=\sum_{i=1}^{\omega -1}v^{Τ}w_{i}+v^{Τ}w_{\geq \omega }=\sum_{i=1}^{\infty }v^{Τ}w_{i}=v^{Τ}\sum_{i=1}^{\infty }w_{i}=v^{Τ}w.$$

Suppose now that we are interested in the age‐specific sensitivity of the natural logarithm of the eigenvalue λ of $\tilde{A}$. Since the blocks $U_{j}$ and $F_{j}$ for j<ω contain the demographic rates specific to individuals of age j, we rewrite the matrix $\tilde{A}$ as

(A17)
$$\tilde{A}\left(\theta_{j}\right)=\left(\begin{array}{ccccccc} F_{1} & F_{2} & \ldots & F_{j}\left(\theta_{j}\right) & \ldots & \ldots & F_{\omega }\\ U_{1} & & & & & & \\ & U_{2} & & & & & \\ & & \ddots & & & & \\ & & & U_{j}\left(\theta_{j}\right) & & & \\ & & & & \ddots & & \\ & & & & & U_{\omega -1} & U_{\omega } \end{array}\right),$$

to highlight that only demographic parameters for individuals of age j depend on $\theta_{j}$. Using (while somewhat abusing notation) the general result of Caswell (1978) in Equation 2 of the main text, Equation A16 and the results above about $\tilde{w}$ and $\tilde{v}$, we have

(A18)
$$\frac{\partial \ln\lambda }{\partial \theta_{j}}=\frac{{\tilde{v}}^{Τ}\frac{\partial \tilde{A}}{\partial \theta_{j}}\tilde{w}}{\lambda {\tilde{v}}^{Τ}\tilde{w}}=\frac{v^{Τ}\frac{\partial F_{j}}{\partial \theta_{j}}w_{j}}{\lambda v^{Τ}w}+\frac{v^{Τ}\frac{\partial U_{j}}{\partial \theta_{j}}w_{j}}{\lambda v^{Τ}w}=\frac{v^{Τ}\frac{\partial A_{j}}{\partial \theta_{j}}w_{j}}{\lambda v^{Τ}w},$$

which coincides with Equation A11 in the main text. This proves the equivalence of the two approaches.

### A.3 Derivation of Equation 12

Here, we derive Equation 12 in the main text by adapting the original derivation by Tuljapurkar (1990), with later extension by Caswell (2005), of Equation 6 in the main text. A derivation of Equation 6 that is more rigorous, yet more complex, than that in Tuljapurkar (1990) concerning limit interchanges is in Steinsaltz et al. (2011). For simplicity, we here adapt the original derivation of Tuljapurkar (1990) as presented in Caswell (2001, sec. 14.4.1) to obtain Equation 12.

We introduce four quantities. First, the time‐specific stage distribution

(A19)
$$w\left(t+1\right)=\frac{A\left(t\right)w\left(t\right)}{e^{Τ}A\left(t\right)w\left(t\right)},$$

where e is a vector of 1s. Second, the time‐specific growth

(A20)
$$\lambda_{t}=e^{Τ}A\left(t\right)w\left(t\right).$$

Third, the time‐specific reproductive value vector

(A21)
$$v^{Τ}\left(t\right)=\frac{v^{Τ}\left(t+1\right)A\left(t\right)}{v^{Τ}\left(t+1\right)A\left(t\right)e},$$

Fourth, the time‐specific backward growth of reproductive value

(A22)
$$\eta_{t}=v^{Τ}\left(t\right)A\left(t-1\right)e.$$

Then, we recall from Tuljapurkar (1990, Eq. 11.2.3) that the stochastic growth rate in Equation 4 in the main text can be expressed as

(A23)
$$\ln\lambda_{s}=\lim_{L\to \infty }\frac{1}{L}\ln\left[v^{Τ}\left(L\right)A\left(L-1\right)A\left(L-2\right)\ldots A\left(0\right)w\left(0\right)\right],$$

where $w\left(0\right)$ and $v\left(L\right)$, that is, the initial stage distribution and the final reproductive value vector, are two independent vectors each having arbitrary nonnegative components that add up to 1. We let

(A24)
$$\beta_{L}=v^{Τ}\left(L\right)A\left(L-1\right)A\left(L-2\right)\ldots A\left(0\right)w\left(0\right),$$

be the quantity within brackets in Equation A23.

Initially, we consider an age‐independent perturbation. Repeatedly using Equations (A19), (A20), (A21), (A22), we write $\beta_{L}$ by making explicit the contribution of projection of the population stage distribution at time t toward the computation of the stochastic growth rate

(A25)
$$\beta_{L}=\left(\prod_{i=0}^{t-1}\lambda_{i}\right)\left(\prod_{i=t+2}^{L}\eta_{i}\right)v^{Τ}\left(t+1\right)A\left(t\right)w\left(t\right).$$

We first consider the effect of a time‐specific, age‐independent change in $\beta_{L}$. In particular, we assume that the projection matrix $A\left(t\right)$ containing demographic rates at time t depends on some parameter $x_{t}$ with current value $x_{t}^{*}$ so that we write this matrix as $A\left(t\right)=A\left(x_{t}^{*},t\right)$. Note that $\beta \left(x_{t}\right)$ also is a function of $x_{t}$. Using Equation A25, the sensitivity of $\beta_{L}$ to $x_{t}$ at $x_{t}^{*}$ is

(A26)
$$\begin{array}{ll} {\left.\frac{\partial \beta_{L}}{\partial x_{t}}\right|}_{x_{t}=x_{t}^{*}} & {\left.=\left(\prod_{i=0}^{t-1}\lambda_{i}\right)\left(\prod_{i=t+2}^{L}\eta_{i}\right)v^{Τ}\left(t+1\right)\frac{\partial A\left(x_{t},t\right)}{\partial x_{t}}\right|}_{x_{t}=x_{t}^{*}}w\left(t\right) \end{array}$$

Suppose now that the change is no longer time specific and all matrices in $\beta_{L}$ may depend on the parameter x. The sensitivity of $\beta_{L}$ to x at $x^{*}$ is the sum of time‐specific sensitivities each having the same form as Equation A26. Thus,

(A27)
$$\begin{array}{ll} {\left.\frac{\partial \beta_{L}}{\partial x}\right|}_{x=x^{*}} & {\left.=\sum_{t=0}^{L-1}\left(\prod_{i=0}^{t-1}\lambda_{i}\right)\left(\prod_{i=t+2}^{L}\eta_{i}\right)v^{Τ}\left(t+1\right)\frac{\partial A\left(x,t\right)}{\partial x}\right|}_{x=x^{*}}w\left(t\right) \end{array}$$

Using Equations (A19), (A20), (A21), (A22), (A23), (A24), (A25) and A27, the sensitivity of $\ln\lambda_{s}$ to x at $x^{*}$ then is

(A28)
$$\begin{array}{ll} {\left.\frac{\partial \ln\lambda_{s}}{\partial x}\right|}_{x=x^{*}} & {\left.=\lim_{L\to \infty }\frac{1}{L\beta_{L}\left(x^{*}\right)}\sum_{t=0}^{L-1}\left(\prod_{i=0}^{t-1}\lambda_{i}\right)\left(\prod_{i=t+2}^{L}\eta_{i}\right)v^{Τ}\left(t+1\right)\frac{\partial A\left(x,t\right)}{\partial x}\right|}_{x=x^{*}}w\left(t\right)\\ & =\lim_{L\to \infty }\frac{1}{L}\sum_{t=0}^{L-1}\frac{{\left.\left(\prod_{i=0}^{t-1}\lambda_{i}\right)\left(\prod_{i=t+2}^{L}\eta_{i}\right)v^{Τ}\left(t+1\right)\frac{\partial A\left(x,t\right)}{\partial x}\right|}_{x=x^{*}}w\left(t\right)}{\left(\prod_{i=0}^{t-1}\lambda_{i}\right)\left(\prod_{i=t+2}^{L}\eta_{i}\right)v^{Τ}\left(t+1\right)A\left(x^{*},t\right)w\left(t\right)}\\ & =\lim_{L\to \infty }\frac{1}{L}\sum_{t=0}^{L-1}\frac{{\left.v^{Τ}\left(t+1\right)\frac{\partial A\left(x,t\right)}{\partial x}\right|}_{x=x^{*}}w\left(t\right)}{\lambda_{t}v^{Τ}\left(t+1\right)w\left(t+1\right)}, \end{array}$$

where we have assumed the validity of interchange of limits, that is, the time limit and the derivative with respect to x. Equation A28, which is due to a variation of Caswell (2005) on the original result of Tuljapurkar (1990), corresponds to Equation 6 in the main text.

We now consider age‐ and time‐specific changes in demographic parameters. We use the fact that Equation A25 singles out the role of demographic projection at time t in the computation of $\ln\lambda_{s}$ to proceed similarly to Section A.1 and write

(A29)
$$\beta_{L}=\left(\prod_{i=0}^{t-1}\lambda_{i}\right)\left(\prod_{i=t+2}^{L}\eta_{i}\right)v^{Τ}\left(t+1\right)\sum_{k=1}^{M+t}A_{k}\left(t\right)w_{k}\left(t\right),$$

where M is the maximum age in the initial population, that is, at t=0, and $A_{k}\left(t\right)=A\left(t\right)$, while the vector $w_{k}\left(t\right)$ is the fraction of the stage distribution of age k at t so that

(A30)
$$\sum_{k=1}^{M+t}w_{k}\left(t\right)=w\left(t\right).$$

Equation A29 makes conspicuous the demographic projection of each age class in the population at t. Similarly to the previous section and above, we model time‐ and age‐specific dependency of demographic rates by assuming that the matrix $A_{j}\left(t\right)$ depends on some parameter $x_{j,t}$ with current value $x_{j,t}^{*}$ so that $A_{j}\left(x_{j,t}^{*},t\right)=A_{k}\left(t\right)$ for k≠j. The sensitivity of $\beta_{L}$ to $x_{j,t}$ at $x_{j,t}^{*}$ is

(A31)
$$\begin{array}{ll} {\left.\frac{\partial \beta_{L}}{\partial x_{j,t}}\right|}_{x_{j,t}=x_{j,t}^{*}} & {\left.=\left(\prod_{i=0}^{t-1}\lambda_{i}\right)\left(\prod_{i=t+2}^{L}\eta_{i}\right)v^{Τ}\left(t+1\right)\frac{\partial A_{j}\left(x_{j,t},t\right)}{\partial x_{j,t}}\right|}_{x_{j,t}=x_{j,t}^{*}}w_{j}\left(t\right) \end{array}$$

Proceeding as in Equations A27 and A28 to remove time specificity and look at the sensitivity of $\ln\lambda_{s}$ to $x_{j}$ at $x_{j}^{*}$, we find that

(A32)
$$\begin{array}{l} {\left.\frac{\partial \ln\lambda_{s}}{\partial x_{j}}\right|}_{x_{j}=x_{j}^{*}}=\lim_{L\to \infty }\frac{1}{L}\sum_{t=0}^{L-1}\frac{{\left.v^{Τ}\left(t+1\right)\frac{\partial A_{j}\left(x_{j},t\right)}{\partial x_{j}}\right|}_{x_{j}=x_{j}^{*}}w_{j}\left(t\right)}{\lambda_{t}v^{Τ}\left(t+1\right)w\left(t+1\right)}, \end{array}$$

which corresponds to Equation 12 in the main text.

### A.4 Derivation of Equations 13 and 14

As in the previous sections, let $x_{j}$ be a parameter with current value $x_{j}^{*}$ upon which the demographic rates of individuals aged j depend. We start by decomposing Equation 3 in the main text, where projection is via the time‐dependent matrix $A\left(t\right)$, as we did for Equation 1 in the main text to get to Equation 10 in the main text. Thus, we obtain

(A33)
$$n\left(t+1\right)=\sum_{\begin{array}{c} k=1\\ k\neq j \end{array}}^{M}A_{k}\left(t\right)n_{k}\left(t\right)+A_{j}\left(t,x_{j}^{*}\right)n_{j}\left(t\right).$$

where *M* is some maximum age. This equation expresses population projection for each age class at t. Recall that $A_{j}\left(t,x_{j}^{*}\right)=A_{k}\left(t\right)=A\left(t\right)$ with k≠j. However, Equation A33 shows that if the population fraction aged j at some point depends on $x_{j}$ (right hand side), then the whole population at the next time point (left hand side) depends on $x_{j}$ as well. Hence, we can write Equation A33 more generally as

(A34)
$$n\left(t+1,x_{j}\right)=\sum_{\begin{array}{c} k=1\\ k\neq j \end{array}}^{M}A_{k}\left(t\right)n_{k}\left(t,x_{j}\right)+A_{j}\left(t,x_{j}\right)n_{j}\left(t,x_{j}\right).$$

Taking then the derivative with respect to $x_{j}$,

(A35)
$$\begin{array}{ll} \frac{\partial n\left(t+1,x_{j}\right)}{\partial x_{j}} & =\sum_{\begin{array}{c} k=1\\ k\neq j \end{array}}^{M}A_{k}\left(t\right)\frac{\partial n_{k}\left(t,x_{j}\right)}{\partial x_{j}}+A_{j}\left(t,x_{j}\right)\frac{\partial n_{j}\left(t,x_{j}\right)}{\partial x_{j}}+\frac{\partial A_{j}\left(t,x_{j}\right)}{\partial x_{j}}n_{j}\left(t,x_{j}\right), \end{array}$$

and evaluating this derivative at $x_{j}=x_{j}^{*}$, when $A_{j}\left(t,x_{j}^{*}\right)=A_{k}\left(t\right)=A\left(t\right)$ with k≠j

(A36)
$$\begin{array}{ll} {\left.\frac{\partial n\left(t+1,x_{j}\right)}{\partial x_{j}}\right|}_{x_{j}=x_{j}^{*}} & =\sum_{\begin{array}{c} k=1 \end{array}}^{M}A_{k}\left(t\right){\left.\frac{\partial n_{k}\left(t,x_{j}\right)}{\partial x_{j}}\right|}_{x_{j}=x_{j}^{*}}+{\left.\frac{\partial A_{j}\left(t,x_{j}\right)}{\partial x_{j}}\right|}_{x_{j}=x_{j}^{*}}n_{j}\left(t,x_{j}^{*}\right)\\ & =A\left(t\right)\sum_{\begin{array}{c} k=1 \end{array}}^{M}{\left.\frac{\partial n_{k}\left(t,x_{j}\right)}{\partial x_{j}}\right|}_{x_{j}=x_{j}^{*}}+{\left.\frac{\partial A_{j}\left(t,x_{j}\right)}{\partial x_{j}}\right|}_{x_{j}=x_{j}^{*}}n_{j}\left(t,x_{j}^{*}\right)\\ & =A\left(t\right){\left.\frac{\partial n\left(t,x_{j}\right)}{\partial x_{j}}\right|}_{x_{j}=x_{j}^{*}}+{\left.\frac{\partial A_{j}\left(t,x_{j}\right)}{\partial x_{j}}\right|}_{x_{j}=x_{j}^{*}}n_{j}\left(t,x_{j}^{*}\right), \end{array}$$

which corresponds to Equation 14 in the main text.

### A.5 Cohort erosion by death

Under standard assumptions in demography about A, we have that λ is the Perron root of this matrix and the corresponding eigenvectors v and w are entrywise positive. Hence, from Equation 2, $\partial \ln\lambda /\partial a_{i,j}=v_{i}w_{j}/\left(v^{Τ}w\right)>0$ so that λ is an increasing function of the entries of A. Hence, we have that $\rho \left(\lambda^{-1}U\right)<\rho \left(\lambda^{-1}A\right)=1$, where ρ is the spectral radius because U≤A entrywise with at least some strict inequality. Therefore, $\lambda^{-1}U$ converges to the zero matrix. From Equation 16, $w_{j+1}={\left(\lambda^{-1}U\right)}^{j}w_{1}$ with j≥1 tends to the zero vector as j goes to infinity. We refer to this as the fact that death eventually completely erodes any newborn cohort.

### A.6 Derivation of Equation 21

Using Equation 16 in the main text, we can rewrite Equation 18 in the main text as

(A37)
$$\varphi \left(\mu_{j}\right)=\frac{v^{Τ}w_{j+1}}{v^{Τ}w}.$$

We then recall the left dominant eigenvector equation for reproductive values

(A38)
$$v^{Τ}=\lambda^{-1}v^{Τ}A.$$

Multiplying $w_{j}$ on the right of both sides of Equation A38, using the decomposition A=U+F and Equation 16 from the main text

(A39)
$$v^{Τ}w_{j}=\lambda^{-1}v^{Τ}{\mathrm{Aw}}_{j}=\lambda^{-1}v^{Τ}{\mathrm{Uw}}_{j}+\lambda^{-1}v^{Τ}{\mathrm{Fw}}_{j}=v^{Τ}w_{j+1}+\lambda^{-1}v^{Τ}{\mathrm{Fw}}_{j}.$$

Let α be the first age at which reproduction is possible. This is the shortest path length from any offspring stage to any reproductive stage in the graph of the transition matrix U (Cochran & Ellner, 1992). Offspring stages are those corresponding to the nonzero rows of the fecundity matrix F, while reproductive stages are those corresponding to the nonzero columns of F. All components of $w_{j}$ corresponding to reproductive stages are equal to zero for 1≤j<α. Some of the components of $w_{j}$ corresponding to reproductive stages are greater than zero for j≥α. Then, we have that $\lambda^{-1}v^{Τ}{\mathrm{Fw}}_{j}=0$ for j<α and $\lambda^{-1}v^{Τ}{\mathrm{Fw}}_{j}>0$ for j≥α. As a consequence, we can establish the following inequalities between the left‐most side of Equation A39 and the first term in the right‐most side of this equation

(A40)
$$\begin{array}{ll} v^{Τ}w_{j} & =v^{Τ}w_{j+1},\;j<\alpha ,\\ v^{Τ}w_{j} & >v^{Τ}w_{j+1},\;j\geq \alpha . \end{array}$$

Dividing through the second line of Equation A40 by $v^{Τ}w$ and using Equation A37, we obtain Equation 21 in the main text.

### A.7 More than one age‐specific parameter

In the main text, we have explored the sensitivity of population growth to a single age‐specific parameter. Here, we give formulas for the sensitivity of population growth to multiple age‐specific parameters. Suppose that demographic rates for individuals aged j are a function of a vector

(A41)
$$\theta \left(j\right)=\left(\begin{array}{c} \theta_{1}\left(j\right)\\ \theta_{2}\left(j\right)\\ \vdots \\ \theta_{p}\left(j\right) \end{array}\right)$$

of p age‐specific parameters. We want to obtain the sensitivity of (the natural logarithm of) stable population growth λ, which is the dominant eigenvalue of the m×m stage‐classified projection matrix A, to this vector. We recall that $A_{j}$ is the projection matrix for individuals of age j. We adopt the formalism of matrix calculus introduced to ecology by Caswell (2007, 2009), see there for more details. In this formalism, the $\mathrm{vec}\left(•\right)$ operator takes a matrix as argument and returns a column vector containing the matrix columns stacked one above the other, the ${\mathrm{vec}}^{Τ}\left(•\right)$ operator takes a matrix as argument and returns the transpose of a column vector containing the matrix columns stacked one above the other and ⊗ is the Kronecker product. Adapting this formalism to our case, the generalization of Equation 11 in the main text to multiple age‐specific parameters is

(A42)
$$\frac{d\ln\lambda }{d\theta^{Τ}\left(j\right)}=\frac{d\ln\lambda }{{\text{dvec}}^{Τ}\left(A_{j}\right)}\frac{\text{dvec}\left(A_{j}\right)}{d\theta^{Τ}\left(j\right)}=\frac{w_{j}^{Τ}⊗v^{Τ}}{\lambda v^{Τ}w}\frac{\text{dvec}\left(A_{j}\right)}{d\theta^{Τ}\left(j\right)}$$

where $d\lambda /d\theta^{Τ}\left(j\right)$ is a 1×p vector with component i equal to the partial derivative of λ with respect to component i of $\theta \left(j\right)$, $d\ln\lambda /{\text{dvec}}^{Τ}\left(A_{j}\right)$ is a $1\times m^{2}$ vector with $\partial \lambda /\partial {\left(A\right)}_{k,l}$ as component $k+\left(l-1\right)m$, while $w_{j}$, w and v are, as in the main text, the stable fraction of the stage distribution aged j (see Equation 16 in the main text), the stable stage distribution (right dominant eigenvector), and the reproductive value vector (left dominant eigenvector) of A, respectively. The partial derivative of the $\left(k,l\right)$‐entry of $A_{j}$ with respect to component s of $\theta \left(j\right)$ is found as the $\left(k+\mathrm{ml}-m,s\right)$ entry of the $m^{2}\times p$ matrix $\text{dvec}\left(A_{j}\right)/d\theta^{Τ}\left(j\right)$.

Similarly, Equation 12 in the main text generalizes to

(A43)
$$\frac{d\ln\lambda_{s}}{d\theta^{Τ}\left(j\right)}=\lim_{L\to \infty }\frac{1}{L}\sum_{t=0}^{L-1}\frac{w_{j}^{Τ}\left(t\right)⊗v^{Τ}\left(t+1\right)}{\lambda_{t}v^{Τ}\left(t+1\right)w\left(t+1\right)}\frac{\text{dvec}\left(A_{j}\left(t\right)\right)}{d\theta^{Τ}\left(j\right)},$$

while Equations 13 and 14 in the main text generalize to

(A44)
$$\frac{d\ln\lambda_{t}}{d\theta^{Τ}\left(j\right)}=\frac{e^{Τ}}{N\left(t+1\right)}\frac{\partial n\left(t+1\right)}{\partial \theta^{Τ}\left(j\right)}-\frac{e^{Τ}}{N\left(t\right)}\frac{\partial n\left(t\right)}{\partial \theta^{Τ}\left(j\right)},$$

where $\partial n\left(t\right)/\partial \theta^{Τ}\left(j\right)$ in Equation A44 is a m×p matrix with $\left(k,l\right)$‐entry equal to $\partial n_{k}\left(t\right)/\partial \theta_{l}\left(j\right)$, which is computed via the recursion

(A45)
$$\frac{dn\left(t+1\right)}{d\theta^{Τ}\left(j\right)}=\left(n_{j}^{Τ}\left(t\right)⊗I_{m}\right)\frac{\text{dvec}\left(A_{j}\left(t\right)\right)}{d\theta^{Τ}\left(j\right)}+A\left(t\right)\frac{dn\left(t\right)}{d\theta^{Τ}\left(j\right)}.$$

with $I_{m}$ the m×m identity matrix.

### A.8 Perturbation pattern of fecundities

We could substitute $\mathrm{sgn}\left(F\right)$ with another matrix with a different pattern of 0s and 1s, that is, representing a different way of additively perturbing fecundity, and still obtain a null sequence. These alternative perturbation patterns correspond to what is deemed biologically realistic in a specific context. For example, one can also consider the possibility of additively incrementing all entries in those rows of $F_{j}$ that contain at least one positive entry. Different nonzero rows of $F_{j}$ identify different offspring stages, where offspring can differ, for example, in their size at birth, that are born to parents aged j. Altering all entries in one of these rows for sensitivity analysis means including in the analysis the effect of altering the fecundity of all stages. But there may be stages that, for biological reasons, cannot be reproductive, for example, a stage comprising individuals of too small size. Moreover, not all reproductive stages may be able to produce all types of offspring, for example, a reproductive stage with small‐sized individuals may not produce large‐sized offspring. For these reasons, we prefer to only look at the effect of additive increments in already existing fecundities.

### A.9 Derivation of Equation 22

First, we derive the sensitivity $\varphi \left(\mu_{k,j}\right)$ of lnλ to a proportional change of the same magnitude exclusively in the entries of $U_{j}=U$ that are in column k. By a reasoning analogous to that brought us to Equation 18 in the main text

(A46)
$$\begin{array}{ll} \varphi \left(\mu_{k,j}\right) & =\frac{v^{Τ}{\mathrm{UE}}_{k,k}w_{j}}{\lambda v^{Τ}w}=\frac{\sum_{i}v_{i}u_{i,k}w_{k,j}}{\lambda v^{Τ}w}=w_{k,j}\underset{=b_{k}}{\underbrace{\frac{\sum_{i}v_{i}u_{i,k}}{\lambda v^{Τ}w}}}, \end{array}$$

where $E_{k,k}$ is a matrix of the same dimensions as U and with $\left(k,k\right)$ entry equal to 1 and 0 everywhere else. Then, we derive the sensitivity $\varphi \left(m_{j,k}\right)$ of lnλ to an additive change of the same magnitude exclusively in the positive entries of $F_{j}$ that are in column k assuming that this column is not zero. Adapting the reasoning that led us to Equation 19 in the main text,

(A47)
$$\begin{array}{ll} \varphi \left(m_{k,j}\right) & =\frac{v^{Τ}\mathrm{sgn}\left(F\right)E_{k,k}w_{j}}{\lambda v^{Τ}w}=\frac{\sum_{i}v_{i}\mathrm{sgn}\left(f_{i,k}\right)w_{k,j}}{\lambda v^{Τ}w}=w_{k,j}\underset{=c_{k}}{\underbrace{\frac{\sum_{i}v_{i}\mathrm{sgn}\left(f_{i,k}\right)}{\lambda v^{Τ}w}}}. \end{array}$$

## APPENDIX B NUMERICAL SIMULATIONS

We made use of the data of Davison et al. (2010) to numerically illustrate a theoretical idea discussed in Section 2.3 of the main text. There, we noted that two initial populations with different age and stage distributions will eventually converge to a common time‐varying age distribution when the same sequence of projection matrices is applied to both populations. Figure B1 gives an example of this phenomenon. As it can be seen, convergence is quite fast and can occur within a few steps of demographic projection.
